# Chemical Routes to Primitive Membranes: Prebiotic Lipid Formation at the Origin of Life

**DOI:** 10.3390/life16030497

**Published:** 2026-03-18

**Authors:** Anastasiia Shvetsova, Michele Fiore

**Affiliations:** 1 Institut de Chimie et Biochimie Moléculaires et Supramoléculaires UMR5246, Claude Bernard Lyon 1, Université de Lyon, 69621 Villeurbanne, France; 2 Laboratoire de Planétologie et Géosciences UMR6112, Nantes Université, 44000 Nantes, France

**Keywords:** amphiphiles, condensing agents, Hadean Earth, hydrothermal pools, hydrothermal vents, lipids synthesis, origin of life studies, phosphorylation of alcohols, prebiotic systems chemistry

## Abstract

The origin of life is, to the best of our knowledge, impossible to imagine without the formation of complex prebiotic biomolecules such as RNA, DNA, proteins and lipids. Lipids play a crucial role in the spontaneous formation of cell membranes, which are responsible for cell integrity, compartmentalization, selective permeability, and providing a microenvironment for biochemical reactions. The goal of the current work is to summarize the current state of the art regarding the abiotic formation of membrane building blocks, such as glycerol, fatty acids, and their phosphorylated version as phospholipid precursors. We describe the necessity of a systems chemistry approach for the complexification and expansion of the prebiotic network, enabling the formation of several membranogenic precursors. We also discuss prebiotic pathways for phosphorylation and acylation that could lead to phospholipid availability in hydrothermal environments and on the early Earth surface. We conclude with the possible spontaneous vesiculation of these molecules as a primitive version of the cell membrane. Thus, we present a comprehensive perspective on prebiotic vesicle formation, starting from simple molecules and developing until the self-assembly of vesicles.

## 1. Introduction

Nature is an open system (within our planet), and the chemistry on the early Earth was most likely “messy,” with numerous starting chemicals present at the same time. It makes tracking the prebiotic reactions that led to the origin of life very complicated. The oldest, most certain fossils were dated ≈ 3.4 Ga old, located in stromatolitic deposits of the Strelley Pool Formation of the Pilbara Craton (Western Australia) [1]. However, the potentially habitable environments for life emergence could just as likely be as early as 4.1–4.3 billion years ago [2]. Life as we know it is based on biopolymers: Ribonucleic acid (RNA) and deoxyribonucleic acid (DNA) carry and transfer genetic information; proteins are essential for metabolism and cellular functioning; and phospholipids form the cellular barrier as a bilayer membrane.

It is a common opinion that the synthesis of those biotic polymers and molecules could take place on the Earth’s surface [3,4,5] and/or in hydrothermal vents [6,7,8]. Additionally, the organic material could be delivered by impact bombardment [9,10,11].

Some of the small organic molecules, such as formaldehyde, methanol, simple amino acids (glycine, alanine and aspartic acid), sugars (glycolaldehyde and glyceraldehyde), pyrimidine and purine bases, and other molecules, were also found in the interstellar medium or in comets and meteorites [10,11,12]. Those materials could also potentially contribute to the organic precursor inventory on the early Earth [13]. Therefore, prebiotic systems chemistry needs to take into consideration all plausible directions and cannot exclude any possible chemical pathways [14].

This review focuses on lipid precursors essential for the formation of protocell membranes. Life as we know it is organized in cellular form, with the membrane serving as the fundamental boundary separating living systems from the non-living environment. This boundary enables the maintenance of a self-sustaining system while providing protection from external stresses [15]. Biological membranes consist of bilayers of amphiphilic phospholipids, in which each molecule comprises a hydrophilic headgroup and two hydrophobic, nonpolar lipid tails. Although membrane composition varies across living organisms, the core structural motif is conserved and typically consists of two fatty acid chains esterified to a phosphorylated glycerol backbone, with diverse headgroups such as hydrogen, ethanolamine, choline, and others [16]. The absolute configuration at the carbon *sn*-2 of the glycerol backbone is opposite between phospholipid esters and ethers, as we have summarized in different previous publications [17,18,19,20]. Accordingly, some membrane properties are different between phospholipid esters and ethers, as reported elsewhere [21].

### Two-Lipid Tail and the Bifurcation at the Origin of Compartmentalized Life

In recent decades, Koga [22,23] and Lombard [24,25,26,27] have advanced the hypothesis that life emerged in prebiotic microenvironments without encapsulation thanks to the synthesis of biotic polymers (before being proteins and RNA) that formed on layers of minerals and in hydrothermal conditions [6,7,28,29,30,31,32,33]. This hypothesis was strengthened by the theoretical studies of Wächtershäuser [25,26,27]. These hypotheses are plausible, but poor evidence proves that metabolisms can exist without the encapsulation, or at least in the form of life as we know it, the cell. Another point is the following: Two different cell types, *Eukarya* (and *Bacteria*) and *Archaea*, formed at the bifurcation of life emergence. The difference is essentially made by the typology of phospholipids forming the membranes. The former has a membrane which contains essentially phospholipid ethers, while the latter has a membrane which contains phospholipid esters. This bifurcation occurred in the early stage of life emergence driven by encapsulation. As summarized by one of us a few years ago, although this differentiation could have occurred randomly, the advent of protein determined the co-evolution of those types of cells [18]. We review the proposed origins of lipid membranes by describing the prebiotic precursors of modern membrane lipids. In particular, we discuss precursors for fatty acid ester lipids associated with bacterial and eukaryotic membranes and isoprenoid ether lipids characteristic of archaeal membranes, without focusing on the biological implications of the “lipid divide” problem.

Without delving into the biological aspects in detail, this paper reviews the geochemical environments of early Earth that were conducive to prebiotic synthesis and outlines plausible chemical pathways that could have led to the spontaneous formation of key molecular components of primitive phospholipid membranes, including phospholipid esters and ethers.

## 2. Geochemical Scenario: The Hadean Earth Is a Goldilocks Planet

**Figure 1:**
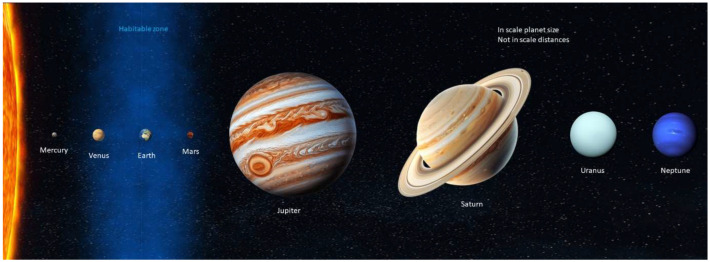
A schematic representation of the solar system. The habitable zone is ideally inside the two blue lines. The drawing is not to scale, and was created with the AI software “Sora 2”.

The presence of liquid water is an essential prerequisite geochemical condition required for life emergence and evolution. By definition, a Goldilocks planet is a planet that falls within a star’s habitable zone (Figure 1), the distance from the star where temperatures allow the existence of liquid water on the orbiting planet’s surface.

The Earth has the prerequisites for the emergence of life as we know it: an optimal distance from the Sun, allowing the presence of liquid water, the availability of essential chemical elements, stable energy sources, and sufficient gravity to retain an atmosphere, long-term environmental stability and geological activity, which is required, e.g., for wet–dry cycles, as discussed below. Furthermore, the presence of a large gaseous planet (i.e., Jupiter, Saturn) protects Earth from catastrophic impacts such as asteroids or comets [34].

Astrophysical observations of exoplanetary systems confirmed the presence of thousands of stars with planets revolving around. Recent discoveries (see Table 1 for remarkable examples) showed that dwarf stars, such as Trappist-1, [35], Proximacentauri b [36] and Kepler-11 [37], possess giant gaseous planets together with Earth-like rocky planets within the habitable zone (HZ). (For a complete report on the exoplanet’s discoveries, see: https://science.nasa.gov/exoplanets/exoplanet-catalog/ accessed on 17 March 2026 at 13h00) Further studies of their atmosphere composition is the next step for understanding the plausibility of their accommodating life or detecting potential biosignatures on their surface.

Exoplanetary worlds are out of reach for humanity at the current state of technology. Therefore, prebiotic simulated conditions are used for bottom-up experiments to simulate plausible conditions that can be found in the habitable zone of Goldilocks planets, including the primordial planet Mars.

The formation of all the essential molecular ingredients for life must have occurred after the large Moon-forming impact [38] and the relatively rapid cooling of the Earth’s crust (between hundreds of thousands or millions of years). The estimation of magma ocean lifetime depends on viscosities, compositions, thermal boundary layer thicknesses, and initial core temperatures.

**Figure 2:**
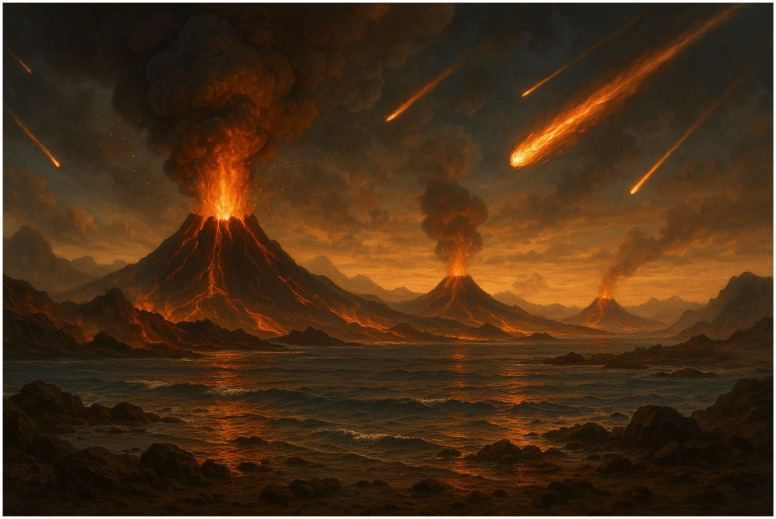
Artistic representation of late Hadean Earth ≈4 Ga ago, created with the AI software “Sora”.

The oldest records of Earth’s history are enclosed in ancient mineral grains, namely zircons, often called “deep-time capsules.” Zircons provide evidence of plate tectonics about 4.3 Ga ago [39], giving us insight into the regime of mantle convection and tectonics on the early Earth. Zircons also suggest the presence of a continental crust at 4.37 Ga ago [40]. At that time, the continental crust was formed and quickly recycled into the mantle. It consisted of cooled mantle rocks interacting with the atmosphere and ocean, influenced by both bombardment and geological processes. Crust–mantle–atmosphere evolution models predict a relatively fast growth of the continental crust, reaching about 80% of its modern volume by the early Archean [41]. This fact enables the presence of different landforms and hydrothermal vents, and the circulation of energy and nutrients from the Earth’s mantle to surface environments (Figure 2).

However, there is no conclusive agreement about the composition and volume of the oceanic and continental crust on the very early Earth due to the significantly limited residual evidence from Hadean history, by definition. The primitive crust was most likely formed of basalts, possibly mixed with some felsic and ultramafic rocks [42]. It sets the geochemical conditions for the emergence of life. In the future, we might acquire more information about Earth’s early history from nearby “models” such as the Moon, Mars, and other extraterrestrial bodies that, in the absence of tectonic activity, preserved older rocks that were recycled on our planet.

During the Hadean, the Moon was much closer to the Earth compared to the present day [43]. This induced stronger tidal effects, which had a broad and significant influence on the Earth’s settings. First, it could contribute to high-amplitude dry–wet cycling in coastal environments. The frequent flooding and draining of tidal zones would create a dynamic environment with alternating periods of wetness and dryness. This would help concentrate chemicals, which could be highly beneficial to the formation of biomolecules. The mixing of seawater and freshwater during tides would cause fluctuations and gradients in pH. Additionally, dynamic tidal effects could provide a source of energy for biochemical reactions and early life forms. The mixing of seawater and freshwater might also create currents and vortices that generate energy. Strong tidal currents would transport organic molecules and thus provide a constant supply of nutrients to the cradle of life.

The presence of immense volumes of liquid water on the Hadean Earth was confirmed by isotopic analyses of oxygen isotopes in zircons [42]. The Sun was significantly fainter (about 70% of today) and much more XUV-active than the present day. This creates the so-called “Faint Young Sun Problem,” i.e., an apparent contradiction between the presence of liquid water on the early Earth and solar activity too low to support moderate temperatures [44,45]. This paradox is resolved when considering different characteristics of the early Earth [46,47]. The composition of the late Hadean atmosphere [48,49,50], which was probably rich in the greenhouse gas carbon dioxide, thereby heated [51,52] the atmosphere together with CH_4_. Additional heat may have arisen from volcanic emissions and tidal warming [47]. Clouds with lower albedo, a low land-to-ocean ratio of the Earth’s surface, and other factors likely contributed to increasing the greenhouse effect in the early atmosphere [46]. Perhaps forthcoming missions to Venus, Titan, and other satellites of the giant planets with heavy atmospheres will help us better understand the environment of the early Earth [53].

Understanding the atmospheric composition is equally important for deciphering the life elements cycle of the Hadean oxygen-free world. There is currently no unified scientific consensus about the composition of the early Earth’s atmosphere. It is commonly considered to consist of large quantities of N_2_/CO_2_/H_2_O [54], with a low amount of oxygen. Atmospheric records during this period confirm the presence of established evaporation and condensation cycles of water [55,56]. The quantity of atmospheric nitrogen is still debated, but it was most probably higher than the present-day level [57]. H_2_ is a very volatile gas and was slowly escaping from the atmosphere [58]. Thus, free O_2_ began to appear much later, after the development of oxygenic photosynthesis and the great oxygenation events [59].

Additionally, meteorite impacts may have converted water to hydrogen and carbon dioxide to methane [60]. In the absence of free oxygen in the atmosphere, the oceans were not only anoxic but also slightly acidic [46]. This allowed for the existence of large amounts of reactive Fe^2+^, which could potentially prompt a variety of prebiotic reactions [61]. The processes of oxidation, corrosion, and weathering of phosphorus-bearing minerals on the Earth’s surface may have concentrated enough phosphate and other microelements for the emergence of life [45,62]. The presence of ferrous phosphorus-containing minerals might have a possible role in phosphorylation reactions.

Under such reducing conditions, atmospheric chemistry may have facilitated the production of nitrogen-reduced compounds, such as hydrogen cyanide H–C≡N and cyanoacetylene H–C≡C–C≡N, which may have accumulated and concentrated in lakes or closed pools. These highly reactive molecules are prone to producing abiotically complex molecules such as nucleobases [44,63]. They are also part of the diverse chemical inventory of organics found in primitive bodies of the solar system, e.g., meteorites, comets, and beyond the solar system in dust clouds. These prebiotic precursors could have been brought to the early Earth by impact bombardment [64]. Amino acids [65], nucleobases [66], sugar-related compounds [67], short aliphatic molecules [68], and macromolecular materials [69] have been found on carbonaceous meteorites [70,71]. In these meteorites, many of the organic molecules are found as complexes of insoluble organic matter, which, under hydrothermal alteration or UV irradiation, produce primary chemical reagents such as ammonia. High-velocity impact shocks might also have provided an additional energy source for the synthesis of several amino acids [72].

Phosphate might have accumulated on the Earth’s surface because of intense impact bombardment [73] and is part of the mandatory chemicals for life in lipids, metabolism coenzymes, RNA, and DNA. However, there is not yet a consensus on the relative amount and chemical composition of extraterrestrial organics delivered to Earth and their implications for the origin of life. The missions to extraterrestrial bodies such as OSIRIS-REx (NASA’s mission to asteroid Bennu) or Hayabusa2 (Japan’s mission to asteroid Ryugu and other asteroids) and analysis of samples in space and after return should provide deeper insights on the nature of organic molecules present on meteorites and comets [74,75].

Among the prebiotic environments favorable to the origin of life, hydrothermal vents attract particular interest. An interesting example is represented by the Lost City system [76,77], which may resemble the early Earth with reactive gases, dissolved elements, and thermal and chemical gradients. Altogether, it comprises a chemically reactive environment favorable to prebiotic synthesis [55,78]. The hot water–rock interactions occurring in hydrothermal flows under pressure may have been a “bridge” between the abiotic and biotic chemistry of primitive life [56].

From the flourishing literature on the origin of life and the environmental conditions that may have witnessed it on early Earth, we can conclude that there are two major settings favored by the origin of life community. The first setting is dry land or shallow-water reservoirs. There, the main building blocks of life result from the organic synthesis from small molecules available on the surface or brought in by meteorite impacts. Reactions proceed with energy provided by UV irradiation, temperature, the presence of minerals, interaction with atmospheric gases, or active organic agents (urea, formamide). The second setting, at hydrothermal vents, places prebiotic reactions on geyser fields or the sea floor. The driving forces are pressure, temperature, concentration, pH, and their gradients, together with the porous surface of chimneys acting as catalysts for chemical reactions. Thus, numerous chemicals such as formamide, sugars, amino acids, and others become available due to the constant flow of material from the vent and other molecules dissolved in water.

There is no conclusive answer as to which early Earth conditions were most favorable for life’s self-assembly. It is also unknown in what precise order prebiotic precursors were introduced into biosynthesis or if other possible forms of proto-life existed. Thus, we take into consideration the range of prebiotically plausible chemical reactions that could fit into the conditions of early Earth described above. We assume there are several alternative pathways to the synthesis of prebiotic precursors under different conditions, each potentially contributing to the origin of life.

Starting with Miller–Urey’s famous experiments in the 1950s, prebiotic synthesis became an exciting research field. It has been greatly advanced, and new approaches have been found to synthesize the essential parts that constitute life. As mentioned before, the primary sources of organic molecules on the early Earth could be extraterrestrial delivery, hydrothermal vents, the atmosphere processed by surface chemistry, or reactions induced by ultraviolet (UV) radiation or lightning strikes, etc.

## 3. The Role of Hydrothermal Fields and Hydrothermal Vents in Early Earth Geochemical Settings

The geology and the chemistry of Earth were completely different before the advent of life, compared to the current conditions. Volcanic heat and hydrothermal sites were important energy sources that could drive the formation of various prebiotic precursors [79,80,81,82]. Compounds like hydrogen cyanide (HCN), formaldehyde (HCHO) and formamide (HCONH_2_) [83,84] were also present in interstellar space, together with water (H_2_O), formic acid (HCOOH), methanol (CH_3_OH) cyanamide (NH_2_CN), acetic acid (CH_3_COOH), acetamide (CH_3_CONH_2_), ethylene glycol (HOCH_2_CH_2_OH) and glycine [85]. Therefore, an infusion of extraterrestrial material (meteorite bombardment, see Figure 2) played a significant role in providing the reservoir of organics by delivery of key prebiotic molecules to the early Earth [86].

**Figure 3:**
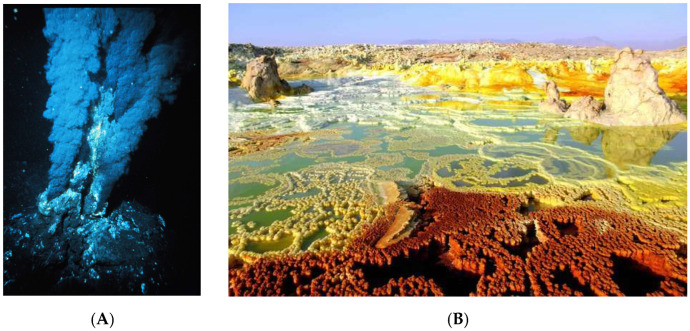
(**A**) Hydrothermal vent on the bottom of the Atlantic Ocean, https://deepoceaneducation.org/resources/hydrothermal-vents/, accessed on 9 March 2026; (**B**) hydrothermal field in Dallo (Africa), https://www.ourbreathingplanet.com/dallol-hydrothermal-field/, accessed on 9 March 2026; all public domain images.

As was discussed before, two different plausible geochemical scenarios for the formation of prebiotic precursors were depicted in the last sixty years. (*The paternity of prebiotic chemistry is nowadays attributed to Alexander Ivanovich Oparin, who in 1924 wrote about the origin of life in his book and intuitively understood that the molecules at the basis of life have originated in volcanic landscapes where energy, minerals and plausibly organic carbon were present at once. Oparin’s book was translated into English from Russian only in 1938* [87].) Both scenarios have a common *leitmotiv*: the presence of liquid water, a source of energy (geothermal but also sunlight/UV radiation), the presence of minerals and small organic molecules. Hydrothermal conditions can be divided into hydrothermal vents (HVs) [7,30,88,89,90] and hydrothermal fields (HFs) [79,82,91]. Hydrothermal vents, also present today at the bottom of the oceans (Figure 3A) are systems whose heat source is the underlying magma or hot water generated by convection currents due to high thermal gradients. HV [92] are alkaline, far-from-equilibrium environments, and until their discovery, they were proposed as sites at which chemical reactions could initiate primitive metabolism involving the reduction in CO_2_ by dissolved H_2_ [7]. The alternatives to HVs are hydrothermal fields (HFs, Figure 3B), known as hydrothermal pools. Damer and Deamer observed that fluctuating hydrothermal pools (FHPs) could be considered as plausibly prebiotic reactors for the synthesis of several key molecules for the development of life, including lipids, nucleic acids and peptides [79]. Notably, a debate is open concerning the role of HVs as chemical reactors for the formation of biotic molecules or their precursors, and only future research can give a definitive reply [93].

In other words, FHPs could be the receptacles of organic, moderately hydrophobic compounds that precipitated, fell into an FHP and accumulated, like a bathtub ring, around its borders at the fluctuating water–atmosphere interface [94]. Therefore, the geochemistry of a young planet Earth was crucial for the abiotic synthesis of such building blocks that are today the constituents of the biomolecules at the basis of life as we know it. A hydrothermal source has several advantages for promoting chemical reactions, and the presence of some minerals, such as clays, can offer better surface contact for the prebiotic synthesis of such molecules [95].

For example, stromatolites—layered mounds, columns, and sheet-like sedimentary rocks that were originally formed by the growth of layer upon layer of cyanobacteria, a single-celled photosynthesizing microbe—represent the microfossil evidence that life started on our planet in the Eoarchean era 4.0–3.6 Gya [96,97,98,99].

## 4. Significance of Systems Chemistry Within Origin of Life Frameworks

In origin of life studies, the bottom-up approach was used to carry out investigations on how the prebiotic formation of different biotic molecules (amino acids and nucleosides, including sugars and phospholipids, without the exclusion of different families of membranogenic compounds) have formed without the intervention of enzymes. To be successful in this approach, the use of activated (i.e., chemical) species is required. As evidenced alongside the following paragraphs, those “activated species” get hydrolyzed before being able to participate in condensation reactions [100]. For example, this aspect has been exhaustively discussed by us in a recent publication on the study of the urea-assisted phosphorylation of alcohols [101].

Furthermore, systems chemistry [14,102,103,104,105,106,107] tries to explain how those molecules, including primitive metabolic intermediates [108], can spontaneously assemble into compartments and develop rudimentary functions such as growth, replication, and selective exchange of materials. Through this double approach, the study of “protocell” research seeks to model the gradual emergence of chemical systems that display increasing autonomy, thereby revealing possible pathways for the transition from abiotic chemistry to the earliest forms of biological organization [109].

Chemically speaking, there is a subtle difference between prebiotically possible and prebiotically plausible. In origin of life studies, plausible prebiotic conditions are those supported by empirical or geological evidence as likely environments on early Earth (or similar worlds), whereas possible prebiotic conditions are broader theoretical scenarios in which the chemical processes needed for life’s emergence could occur, even if they did not necessarily exist historically, in accordance with geologically proven ones. Thus, in our review, we refer only to plausible scenarios, avoiding any possible but implausible scenarios, even if not specified. Different geological settings were favorable (plausible) for prebiotic reactions that led to the synthesis of biologically significant building blocks.

Although the conceptual foundations of prebiotic systems chemistry were established by Oparin [87,110], the first genuinely bottom-up experiment in prebiotic chemistry is attributable to Stanley Miller. Miller observed that the spontaneous formation of amino acids was observed using simple and prebiotically available compounds such as methane, ammonia, water [111,112,113,114,115]. In the years that followed, this pioneering research inspired many other scientists. More than one researcher invested significant resources and devoted years of their career to exploring this area of life sciences, investigating the plausible role of specific building blocks in the formation of various biomolecules under prebiotic conditions, in the absence of any enzyme-driven reactions. In particular, the pioneering work of Eschenmoser & Lowenthal [116] inspired Sutherland to set up a chemical system that simulates a reaction network. As a result, a remarkable result was reached. Precursors of RNA, amino acids, and lipids were prepared in a plausible network. The main actors are the reductive homologation of HCN, where hydrogen sulfide (H_2_S) and UV light provide the reaction mixtures with reducing power. This hypothetical cascade (reaction network) was outlined and termed “cyanosulfidic proto-metabolism” [117].

Less or poorly explored is the field of the abiotic formation of membranogenic compounds. Other researchers have, in the last 50 years, followed the footprints left by Oparin, like Oro [118], Deamer [119,120,121], Powner [122], Monnard [123], Strazewski & Fiore [18,101,124,125], Chen [126], Walde and Luisi [127,128] in the field of systems chemistry of amphiphiles. In addition, Ritson and Sutherland demonstrated an entire reaction network connecting the formation of sugars (tetroses and pentoses), terpenoids and glyceryl phosphate based on reactions with inorganic sulfur species and hydrogen cyanide [129].

One of the most relevant reactions in this field is the phosphorylation of alcohols, a condensation reaction between inorganic phosphate and an alcohol [130]. Strazewski and co-workers made an indisputable contribution, namely, glycerol phosphorylation by thiophosphates in formamide. PSO_3_^3−^ by itself is an effective phosphorylation product with yields of glyceryl monophosphates of about 35% after heating for 10 h at 75 °C. Even lower temperatures are favorable for phosphorylation in the presence of a chemical activator. Phosphorylation with the addition of acrylonitrile or iron cyanate reveals similar yields, but at rt. UV irradiation induces phosphorylation with comparable yields at 40 °C. The effectiveness of thiophosphate can be explained by good leaving group formation through the reaction of H_2_S that facilitates phosphorylation. This example, among others, represents an important step in the development of the systems chemistry approach, which is essential for further progress in understanding the origin of amphiphiles in particular, and the origin of life more broadly [55]. Within the framework of prebiotic systems chemistry, the authors established a systematic investigation of how membranogenic compounds could form under the aforementioned conditions and examined the role these compounds may have played in the emergence of the first protocell-like structures [101].

## 5. Formation of Lipids and Membrane Precursors

### 5.1. Prebiotic Formation of Glycerol

Glycerol plays a functional role as the structural backbone of phospholipids. It is an essential part for lipid precursors of both acylglycerols (lipids lacking the polar head) and phospholipids (possessing the phosphate polar head) [131]. The prebiotic formation of glycerol can be a part of a formose-type reaction in which UV acts as an energy source [117,132,133]. UV is the energy source for the formation of glycerol occurring on interstellar bodies through different chemical processes, including the photolysis of mixtures of CO, NH_3_ in H_2_O at 12 K [134] on ice film of NH_3_, CH_3_OH, HCN in H_2_O at 15 K [135], by the reaction of gas mixture CO, NH_3_, and H_2_O on an aluminum block at 10 K [128], and from ice grains of H_2_O, CH_3_OH, and NH_3_ [117,129,130,131,132,133]. Glycerol is also produced in the cyanosulfidic proto-metabolism largely explored by Sutherland and co-workers from the disproportion of two molecules of glyceraldehyde [117].

### 5.2. Long Chain Formation

UV irradiation is also a good way to obtain *n*-decanoic and *n*-hexadecanoic alcohols from corresponding *n*-alkanes (*n*-RH) with 1-naphthol [136]. Some quantities of monocarboxylic acids were produced in classical Miller–Urey experiments induced by spark discharge (C_2_–C_7_) [137] and semi-corona discharge (C_2_–C_12_) [138] from simple molecules such as methane, ammonia, hydrogen and water [130].

Long-chain fatty acid formation under hydrothermal conditions via Fischer–Tropsch-type (FTT) reactions [6]. In such systems, carbon monoxide, carbon dioxide, or small organic acids can be converted into hydrocarbons and oxygenated products at elevated temperatures in the presence of transition metals or their minerals. For example, heating formic or oxalic acid at 175 °C for 2–3 days produced a mixture of *n*-alkanols, *n*-alkanoic acids, *n*-alkenes, *n*-alkanes, and *n*-alkanones (C_2_–C_35_) [139]. Similarly, the thermal decomposition of iron oxalate (FeC_2_O_4_·2H_2_O) at 400 °C yielded long-chain fatty acids, a process that may be interpreted as generating reactive carbon species capable of undergoing FTT-type chain growth on iron-containing phases [140]. Comparable experiments performed with oxalic acid solutions at 150–250 °C for two days resulted in C_12_–C_33_ lipids, including *n*-alkanols, *n*-alkanoic acids, *n*-alkyl formates, *n*-alkanals, *n*-alkanones, *n*-alkanes, and *n*-alkenes [140,141,142].

Pressure and heat have also been responsible for the production of the same mid– long chain hydrocarbons, including fatty acids and alcohols, as reported by Mayer and co-workers. Analyzing different samples of Archaean quarts collected in western Australia formed between 1 K and 2 K meters, they observed the presence of long-chain aldehydes. The plausible temperature was similar to that of hydrothermal vents (50 °C/100 bar hydrostatic pressure), allowing the possible FTT reaction [143]. The main discovery of this research is long-chain aldehydes [144].

Again, a Fisher–Tropsch-type reaction has occurred on carbonaceous chondrites. It was explored in experiments promoted by meteoritic iron, iron ore and nickel–iron alloy [145]. As an example, fatty acids were produced along with alkanes, alkenes (C_1_–C_25_), aromatic hydrocarbons and others after heating deuterium with carbon monoxide at 200–370 °C for 6–480 h.

Other interesting precursors in terms of membranogenic compounds are dodecanol-1 or geraniol. The formation of dodecan-1-ol, as one of the long-chain alcohol molecules, could be possible in accordance with other methods described above for lipid precursor’s synthesis.

Membranes of Archaea, which are among the oldest microorganisms, are formed of terpenoids such as geraniol, which make them more resistant to temperature. It is possible that primitive or protocells also had such a molecule in their membrane, for which geraniol could be a potential prebiotic precursor [146,147]. One approach is to synthesize geraniol by C_5_ monoprenol condensation in the presence of montmorillonite K-10 at room temperature after 9 days [147]. Other pathways to produce similar molecules, such as squalene [148] from farnesol in the presence of a redox system H_2_S/FeS or as polyprenols [149] from five-carbon homologation of regular prenyl halides, are assisted by two regio- and stereospecific routes to compounds of the polyprenol (dolichol) series. The information regarding this area of research is very limited. One of the few published suggestions is by Ritson & Sutherland [129], who used a prebiotic pathway leading to a monoprenol or even the isoprenoid (terpenoid) carbon skeleton.

### 5.3. Acylation Step and Role of Condensing Agents

**Figure 4:**
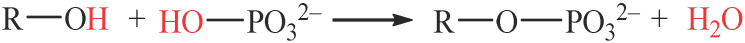
Principle scheme of alcohol’s phosphorylation. The atoms of the starting molecules that formed water as a result of the condensation reaction are highlighted red.

The further step is acylation, which brings together fatty acids and glycerol. This is a dehydration-type chemical reaction that takes water away again. One way to accomplish it is by using a “condensing” agent, i.e., a molecule that will help to take H_2_O away (see Figure 4) [131,150,151].

In his pioneering study, Deamer observed the formation of phosphatidic acids when long-chain aldehydes, glycerol, and inorganic phosphate were mixed in the presence of dicyanamide as a condensing agent (ca) [5], and this is the first example of prebiotic systems chemistry known in the field.

Cyanamide was extensively used as ca by Oró and colleagues [152]. The presence of cyanamide helps to produce acylglycerols from mono-, di-, and tri-palmitoyl glycerol derivatives at 60–100 °C. Rushdi & Simoneit demonstrated lipid formation, as discussed before, and continued work on lipid acylation in hydrothermal conditions [140,141]. Mono- and diacylglycerols were obtained after heating n-heptanoic acid with glycerol at 100 to 250 °C. In similar conditions, alkanoic acids (C_7_–C_16_) and glycerol in water produced mono-, di- and triacylglycerols [142]. Lipid formation is also favorable in the presence of silicic acid and kaolin solution from glycerol and C_12_ fatty acids after incubation for several days at 65 °C (≈3%) [5].

## 6. Phosphorylation of Lipids in Prebiotic and Hydrothermal Conditions

### 6.1. Role of Condensing Agents in Phosphorylation Reactions Under Prebiotic Conditions

Since life is impossible without water and it is a universal solvent for many biological reactions, it is assumed that prebiotic reactions had to happen in water [153,154]. However, looking at the principal reaction of alcohol’s phosphorylation (Figure 4), we instantly see thermodynamic constraints. In the presence of water, hydrolysis is preferred to the formation of phosphoesters. Thus, it was suggested that hot, dry, and/or evaporative conditions are more favorable for phosphorylation.

**Figure 5:**
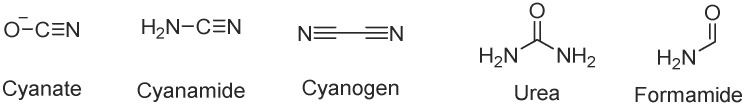
Molecular structures of potential prebiotic condensing agents.

There are several pathways to overcome the water problem. One of them is the assistance of a “condensing” agent. If those molecules are co-localized with alcohol and phosphate sources, it provides a thermodynamic driving force for the phosphorylation reaction by irreversibly eliminating water, thus acting as an efficient condensing agent. Earlier, we reviewed the involvement of those agents for lipid formation and acylation reactions. The molecules known as good condensing agents are cyanate, cyanamide, urea and formamide (Figure 5).

Among the best-known condensing agents is cyanamide and a variety of its precursors and derivatives. Many papers describe the origin of cyanamide on Earth, for example, by UV irradiation of cyanide solutions or electron irradiation of CH_4_, NH_3_ and H_2_O mixtures [155].

The condensing agent not only assists in the interaction of phosphate and alcohol, but in excess, it also promotes the formation of cyclic organic products of phosphorylation [101]. In water, cyanamide expectably produces urea, which is its first hydration derivative. Several publications confirm the plausibility of urea on the early Earth [156,157]. It is assumed that such a molecule could be available in the prebiotic environments described above, such as dry land or evaporative lakes.

**Figure 6:**
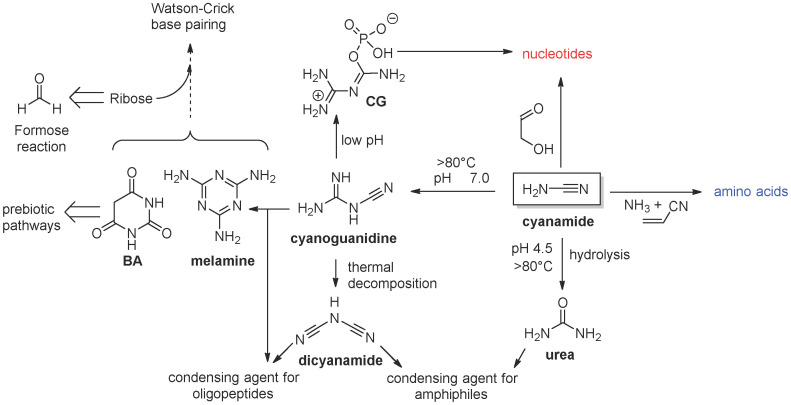
Representation of chemical transformations of cyanamide.

Condensing agents have value not only in phosphorylation but also in many other prebiotic syntheses; an example of a comprehensive summary for cyanamide was suggested by Fiore and Strazewski a few years ago [131], Figure 6.

Adding a condensing agent is sufficient when the energy of the molecule’s hydrolysis matches the energy requirement for phosphorylation. Pasek demonstrated an example of urea-assisted phosphorylation of a nucleoside, where urea hydrolysis (−14.8 kJ/mol) is close to nucleoside phosphorylation (+14.2 kJ/mol) [158]. Therefore, the overcoming of the thermodynamic barrier is possible by producing reaction intermediates between the condensing agent and other reactants. Thus, effective phosphorylation can be achieved in prebiotic environments. Therefore, the literature suggests that the presence of condensing agents enhances the yields of phosphorylation reactions under relatively mild conditions, as reported alongside the nest paragraphs.

### 6.2. Phosphorylation of Glycerol and the Effect of Condencing Agent

To date, no research papers have reported the simultaneous production of phosphate and carboxylic esters within the same environment. However, such a step is essential for the formation of membranogenic phospholipids from fatty acids, glycerol, and a phosphate source. Ideally, this environment would also be conducive to nucleotide formation and their subsequent oligomerisation [159]. The formation of cell membranes depends on both lipid precursors and phosphorylated glycerol derivatives. The phosphorylation of glycerol is more effective under many relatively prebiotic conditions due to its stability, solubility, reactivity and liquid state at the “temperature window” (25–160 °C) of prebiotic chemistry.

**Figure 7:**
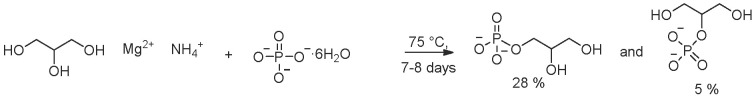
Reaction of glycerol phosphorylation, adapted from Gull et al. [161].

A straightforward experimental procedure for glycerol phosphorylation was demonstrated by Pasek et al. [160]. A water solution of schreibersite with glycerol was heated at 65 °C in anoxic conditions. The major products were inorganic phosphate and phosphite, along with a minority of glyceryl phosphates (2.5%). This experiment confirmed that P-sources suitable for phosphorylation may come from meteorites, and that alcohol can be phosphorylated under the same conditions in water at a mild temperature without additional agents. Another prebiotic mineral, struvite, was tested in a water solution with glycerol [161]. The reaction mixture was heated at 75 °C for 7–8 days. As a result, glyceryl phosphates were formed in yields of about 28% and 5% for the primary and secondary positions, respectively (Figure 7).

Another approach to the phosphorylation and acylation of glycerol was demonstrated by Zhao et al. [162], where impact shock was used to promote the reactions. The authors explored multiple reaction settings using glycerol, sodium octanoate, serpentine, and inorganic phosphates under dry and wet conditions, simulating impact environments at pressures up to 30–35 GPa. It is assumed that intense early Earth bombardment could serve as both an energy source and a supply of starting materials. However, it yielded relatively low product formation, with glyceryl phosphate yields ranging from ~0.1 mol % to a maximum of ~7.5 mol %.

Even though the phosphorylation of glycerol is achievable directly by reacting with a phosphate source, the yields remain low in the absence of additional organic activators. Their presence decreases the activation energy of phosphorylation and increases the yield of the reaction.

Epps et al. studied the phosphorylation of glycerol by ammonium dihydrogen phosphate in the presence of urea or cyanamide [163]. A water solution of glycerol, a condensing agent and NH_4_H_2_PO_4_ was evaporated and heated at 85 °C for 16 h. As a result, glyceryl-1 (and 2) phosphates along with cyclic glyceryl phosphate were obtained. The maximum yield of phosphorylated products was 33% in the reaction with cyanamide. A comparable result in the urea-assisted reaction was achieved only with the addition of NH_4_Cl.

Phosphorylation in a choline chloride/urea [164] eutectic solution was studied for glycerol phosphorylation. The yields of phosphorylated products (the majority are acyclic glyceryl phosphates) were higher in a reaction with inorganic phosphate (65%), struvite (65%) and phosphite (52%) after 7 days of heating at 65 °C. Later, the same research group proposed glycerol as a starting molecule and, at the same time, as a part of the eutectic solution glycerol and choline chloride (2.5:1) to compare it with formamide [165]. The total yields of organic phosphorylated products for all tested phosphates were low: 0, 15 and 15% for NaH_2_PO_4_, cTMP and struvite, respectively; the reaction in formamide was more efficient, resulting in 16, 37 and 35%, respectively. Another factor explored by the authors was the effect of kaolinite and quartz on the reaction intensity. Their presence in both solvents greatly increased the production of phosphorylated products, up to a maximum of, for example, 87–90% in the reaction of struvite in formamide. The authors do not give a definitive answer but suggest an explanation based on the mineral reactivity or catalytic activity of kaolinite and quartz that promotes the reaction.

The phosphorylation of glycerol in eutectic urea/ammonium formate/water [166,167] or choline chloride/urea [164] follows the same trend as nucleoside reactions. The experiment in eutectic urea/ammonium formate/water solution [166,167] of glycerol with Na_2_HPO_4_ at 65 °C after 22 days resulted in cyclic and acyclic glycerol monophosphate and diglycerol mono- and diphosphates, along with several carbamoylated glyceryl derivatives. Unlike nucleosides, lower temperatures were more favorable for the phosphorylation of glycerol. In the absence of urea, reactions were not effective, even at higher temperatures or in the presence of different phosphates.

Maguire et al. [168] demonstrated a prebiotically plausible pathway for the phosphorylation of glycerol and other prebiotic precursors using thermodynamically activated intermediate imidazole phosphate. It is formed in the reaction of cyanate, orthophosphate and imidazole and serves as an effective phosphorylation agent, producing glycerol-1-phosphate and glycerol-2-phosphate in relatively high yields up to 70%.

A key limitation of the cited methods pathway is the small probability of the presence of such a large concentration of cyanate and imidazole or urea/ammonium formate/water agents, which may limit the occurrence of phosphorylation reactions in the early Earth environments. It is worth mentioning that glycerol is a relatively reactive molecule; however, for fatty acids and acylated glycerol derivatives, discussed below, the presence of a condensing agent is mandatory [161].

### 6.3. Phosphorylation of Long-Chain Alcohols Including Decanol and Geraniol

Albertsen et al. reported the formation of *n*-decyl phosphate [123]. It was synthesized from *n*-decanol in a phosphorylation reaction with urea and NH_4_(H_2_PO_4_) at 100 °C. Later, the authors demonstrated the formation of vesicles from the obtained crude mixtures as well as from pure *n*-decyl phosphate. Fiore and co-workers instead worked on undecane-1-ol, obtaining similar results, opening to the “odd number” fatty orthophosphate series. Remarkably, cyanamide was used as a reservoir of urea, as demonstrated via hydrolysis and dimerization under prebiotically plausible conditions [169].

**Figure 8:**
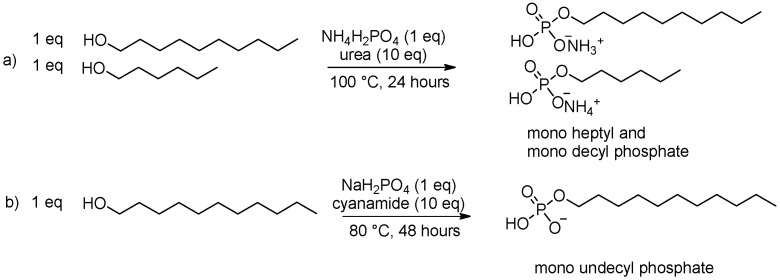
Phosphorylation reaction of alkanols, adapted from Powner and Sutherland [170] and Fiore and co-workers [169].

Powner and Sutherland [170] performed competitive phosphorylation of mixed-length alkanols under the same conditions that they had suggested for nucleotide synthesis [171]. A mixture of ethanol, hexanol, decanol, urea and ammonium dihydrogen phosphate were dissolved in water, dried at 40 °C and heated for 24 hours at 100 °C. The reaction was chemoselective towards decyl- over hexyl-phosphate, with a complete absence of ethyl phosphate (7:3:0, respectively). The authors explained it by the reversibility of phosphorylation and the progressively lower volatility of ethanol, hexanol and decanol (Figure 8).

Archean lipids, hopanoids, carotenoids, sterols, dolichols, ubiquinones are only a few examples of the incredibly diverse and pervasive terpenoid components found in biological membranes. Research in particular about geraniol phosphorylation is an active field of research and its direct phosphorylation and prebiotic plausibility are debated. However, there are several approaches to it.

A theoretical approach by Ourisson [172] envisaged that the phosphorylation of polyprenyl alcohols would work in the same way as other prebiotic alcohols and, at the next step, form vesicles.

**Figure 9:**
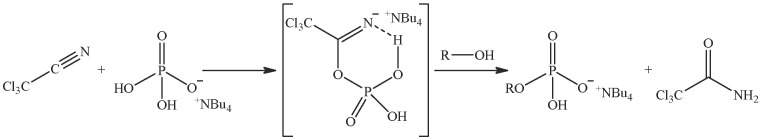
Mechanism of alcohol phosphorylation, as suggested by Lira et al. [173].

Lira et al. [173] suggested the one-pot phosphorylation of alcohols, including geraniol and other starting molecules. They used a direct approach and achieved a 66% yield of geranyl monophosphate in a reaction with tetrabutylammonium dihydrogenphosphate, which is used as the phosphate donor in combination with trichloroacetonitrile as a condensing agent. The reaction mechanism is similar to the phosphorylation by cyanamide described before (Figure 9).

Using the same method of phosphorylation, Pozzi et al. [174] produced monopolyprenyl mono- and diphosphates from geraniol, farnesol, geranylgeraniol, dodecanol and others. As a result, stable vesicles were produced only from the last two alcohols. The failure of geraniol can be explained by its complete solubility in water under the studied conditions. At the same time, stable vesicles detected by optical microscopy were formed from a crude mixture of dodecanol phosphates.

There are several fast and effective approaches for phosphorylating various polyprenols. First, the reaction of trichloroacetonitrile with tetra-n-butylammonium dihydrogen phosphate solubilized in chloroform or acetonitrile [175] has a yield towards a phosphorylated product of 27–87%, depending on the starting molecule. To accomplish effective phosphorylation, at least three mol of phosphate per mol of prenol is required. The method proved to be relevant to a broad variety of α-unsaturated polyprenols: polyprenyl phosphates that can be phosphorylated with medium-to-high yields.

Second is the approach suggested by Keller and Thompson for the preparation of isoprenol diphosphates [176]. The authors present a concise methodology for the efficient synthesis of isoprenoid diphosphates with carbon chain lengths ranging from C_5_ to C_20_. The phosphorylating reagent employed in this process consists of bis-triethylammonium phosphate dissolved in trichloroacetonitrile. The reaction occurs in 15 min, with final yields of phosphorylated product after isolation of 17–35%.

Nakatani et al. suggested a hypothetical approach to the formation of vesicles from polyprenols and glycerol [177]. The phosphorylation of glycerol could be approached by amido-triphosphate or other sources in the presence of Mg^2+^.

The phosphorylation of dodecanol and geraniol was tested in prebiotic conditions in the presence and absence of different condensing agents and their excess with respect to starting alcohol molecules [101]. In the result of heating excess urea with NaH_2_PO_4_ and dodecanol-1 or geraniol, the phosphorylated products were found in trace quantities.

Similar to the biosynthesis of geranylgeranyl-glyceryl phosphate from geranylgeraniol and glyceryl phosphate, the nucleophilic attack of glyceryl phosphate on polyprenyl pyrophosphate could generate polyprenyl-glyceryl phosphates. Polyprenyl-glyceryl phosphates have the potential to form vesicles as well, perhaps when paired with a sufficient quantity of polyprenols. However, the potential for vesicle formation may be limited by the hydrophobic/hydrophilic equilibrium inside the polyprenyl-glyceryl phosphate structure.

### 6.4. Production of Phospholipids and Their Polymerisation

Acylated glyceryl molecules obtained during the condensation reaction induced by silicic acid and kaolin can be phosphorylated under the same conditions [5]. Glycerol, dodecanoate, phosphate and dicyanamide were incubated together for 12 h at 65 °C, resulting in the formation of <1% of phospholipids. Even from such a small purified fraction, the authors managed to produce stable vesicles.

**Figure 10:**
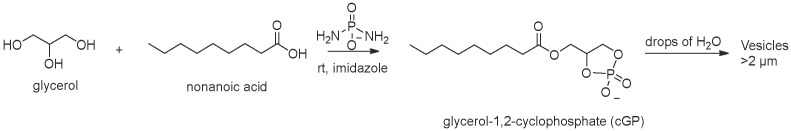
Reaction of one-pot phosphorylation and esterification of glycerol and its vesiculation, adapted from Gibard et al. [178].

Gibard et al. tested the phosphorylation and polymerization of glycerol and fatty acids in both dry and wet–evaporative conditions (water initially is present in the reaction mixture but evaporates eventually during heating) [178]. Diamidophosphate (PO_2_(NH_2_)_2_ was used as a phosphate source and played the role of a condensing agent. The synthesis of glycerol-1,2-cyclophosphate (cGP, 12%) at rt and diglycerol-phosphodiester at 55 °C was achieved. In the next step, the synthesis of phospholipids was attempted. A one-pot experiment of glycerol and nonanoic acid in the presence of diamidophosphate and imidazole led to the formation of a cyclophospholipid (cGP) after several days at rt (Figure 10).

This makes diamidophosphate an interesting potential phosphate source, especially in terms of further polymerisation of nucleotides in water. The latter authors suggest that diamidophosphate could be formed prebiotically from condensed phosphates and facilitate intramolecular phosphorylation by nucleophilic attack of the NH_2_-group in the absence of an additional condensing agent [179].

Fiore, again, demonstrated the phosphorylation and further vesiculation of racemic dioleoylglycerol [125]. It was mixed with cyanamide (or urea) and NH_4_H_2_PO_4_ for 24–48 h at 80 °C in dry or wet–evaporative conditions. As a result, the main product, racemic dioleoylglyceryl phosphate, was obtained with the by-products mono-oleoyl glycerol, mono-oleoylglyceryl phosphate and oleic acid.

**Figure 11:**
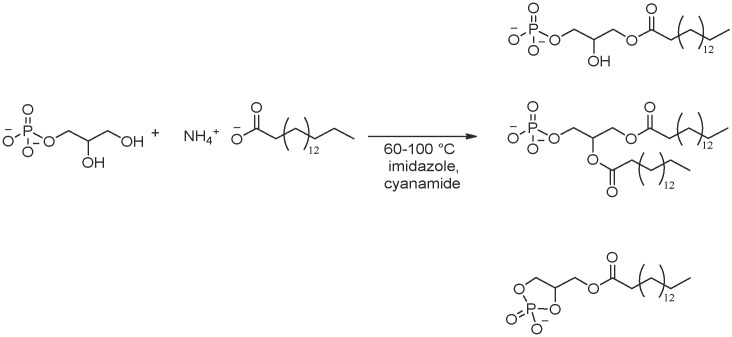
Reaction of glycerol phosphate acylation in the presence of cyanamide, adapted from Epps et al. [152].

Phospholipids can be produced not only by phosphorylating acylated glyceryl derivatives directly but also by glyceryl phosphates reacting with fatty acids. Such an approach to the synthesis of phospholipids was suggested by Epps et al. [152]. In the reaction of glyceryl-1(3)-phosphate with ammonium palmitate and cyanimide, monopalmitoylglycerophosphate (MPGP), dipalmitoylglycerophosphate (DPGP) and monopalmitoyl cyclic glycerophosphate (cMPGP) were synthesized with a maximal conversion of glycerol phosphate of 45% after heating at 60–90 °C for several hours (Figure 11). The average ratio of products is 60% MPGP, 27% DPGP and 13% cMPGP from the top to the bottom in Figure 11.

Bonfio et al. [180] suggested a pathway through heating the acyl imidazolides of fatty acids (C_4_, C_6_, C_8_, C_9_, and C_10_ acyl chains) at 50 °C in water or water–formamide solutions. As a result, libraries of different chain-length mono- and bis-acylated products were produced. Stable membranes were formed only on the basis of C_8_–C_10_ acylglycerol-2-phosphates.

Results of Alexandrova et al. [181] demonstrated that the functionalization of phospholipids can be achieved via ring opening of cyclic glyceryl phosphate. The authors synthesized phospholipids with different head groups, such as choline or lysophospholipids. The highest yields (up to ~22%) were obtained under evaporative conditions at temperatures around 60 °C. In short, there is a prebiotically plausible pathway for lipid phosphorylation and their further spontaneous self-organization into vesicles.

## 7. Spontaneous Vesicle Formation

A defining feature of living systems is the ability to compartmentalize bioactive molecules within bounded spaces. Closed membranes confine, protect internal chemical networks, and can additionally support reactions within their hydrophobic phase [159]. In extant organisms, such compartments are primarily composed of phospholipids, together with proteins, carbohydrates, and other lipid species [106].

Three major classes of prebiotic compartments have been proposed:

1. Lipid-based compartments, formed by self-assembling amphiphiles such as fatty acids or phospholipids.
2. Non-lipid compartments, such as microdroplets produced by α-hydroxy acids (αHAs).
3. Non-bounded systems, such as mineral surfaces, which concentrate molecules without forming enclosed membranes.

It is a common opinion that the encapsulation of biotic molecules occurred into multilayer membranes, as suggested by Deamer [91,120,182,183,184,185], whereas the role of micelles (or the inverse ones) in the origin of life has been poorly explored [186,187]. It is important to mention the role of mono alkyl phosphate [123,169] and fatty acids [188,189,190,191] as a possible prebiotic environment for a “Darwinian” evolution of primitive forms of life [192,193,194].

The spontaneous self-assembly of amphiphilic molecules into closed, bilayer membranes likely represented a key prerequisite for the molecular evolution of protocells. Such compartments could be sufficiently stable to retain macromolecules while remaining compositionally dynamic and selectively permeable to small, reactive molecules [195]. Although modern biological systems universally rely on lipid bilayer membranes, the chemical diversity of the early Earth suggests that primitive living systems may initially have employed alternative boundary structures [121,196].

A living chemical system, as currently understood, consists of an organized network of reactions maintained far from thermodynamic equilibrium through continuous energy input. This network would encompass a wide range of molecular species—including oligonucleotides, peptides, lipids, carbohydrates, and others—whose spatial co-localization is essential for cooperative interactions, emergent properties, and ultimately the emergence of life [106]. Encapsulation gathers these components within a confined volume, thereby exerting a strong influence on their chemical behavior. Confinement increases local concentrations of reactants and catalysts, accelerating reaction rates and enhancing the efficiency of the network. In the absence of confinement, dilution would slow reaction kinetics and prevent the system from being sustained far from equilibrium. Confinement further modulates reaction selectivity and stabilizes transient species, including molecular complexes. In addition, chemical reactions may occur at the compartment boundary itself, contributing to the catalytic capacity of the system [197,198,199]. However, compartmentalization must not imply complete isolation: Sustained chemical activity requires the influx of energy and nutrients and the efflux of waste products. Semi-permeable membranes, analogous to those of modern cells, enable such selective exchange.

In early protocellular systems, identity was most likely encoded in sequence-defined polymers, with oligonucleotides representing the most plausible candidates [106]. Encapsulation is therefore critical for maintaining the physical association between these informational molecules and their corresponding reaction networks, thereby preserving the integrity of the system [19]. The boundary structure itself further contributes to protocellular identity by regulating the selective exchange of materials between the internal milieu and the surrounding environment. For Darwinian evolution to occur, this identity must be capable of replication with occasional variation, generating competing systems subject to selection pressures [192,193,194]. Because protocellular identity is embedded within the encapsulated chemical network, only a compartmentalized structure—comprising both the membrane and its contents—can replicate as an integrated unit and thus undergo evolutionary change. Given the profound effects of confinement on chemical reactivity [198], encapsulation is thought to have arisen at an early stage, leading to the emergence of abiotic systems at the interface between chemistry and biology, commonly referred to as protocells [159,200,201,202,203]. Through gradual evolutionary processes, such protocells likely bridged the transition from non-living chemical systems to the Last Universal Common Ancestor (LUCA). However, reconstructing the nature of the earliest compartments remains highly challenging, as both geological and phylogenetic evidence are unable to probe events at such deep timescales [18].

## 8. Conclusions

The origin of life remains an important subject for understanding the appearance of the first living organisms on our planet, as well as the potential detection or search for biosignals in other exoplanetary systems. However, due to the lack of evidence from early Earth history, many pieces of the puzzle of life are still missing. Among other fundamental questions, such as how the first biomolecules formed and how primitive metabolic networks emerged, the current work focuses on the formation and appearance of the protocell, and thus the cell membrane, upon proven geological conditions on early Earth.

Here we reviewed recent advances in a key step toward understanding the origin of life: membranogenic formation. For the first time, the pathway starting from the formation of prebiotic precursors, such as glycerol and fatty acids, through their complexification via phosphorylation and acylation reactions toward membranogenic precursors and their spontaneous vesiculation is tracked and summarized in a single paper. We highlight the importance of a systems chemistry approach within origin of life research, treating it as an open and complex system.

All suggested experiments are discussed within the framework of the plausibility of the required chemical inventories and geological settings in which these reactions could have occurred on the early Earth. The prebiotic reactions are reviewed under two different environmental conditions: wet–dry cycles on emerged land (tidal zones, shallow lakes) and hydrothermal seafloor vents and geysers.

Each section is illustrated by up-to-date research, aiming to address major questions and challenges related to membrane-forming precursors, such as the phosphorylation problem, the role of condensing agents, and the challenges of spontaneous reactions, among others. This paper provides a clear illustration of current advances in the field and builds a solid foundation for future research in prebiotic membrane chemistry and origin of life studies.

## Figures and Tables

**Table 1:** Summary of the most studied planetary systems. The letters correspond to the planet’s position within the star system.

Planet	System	Distance (ly)	Notes
TRAPPIST-1 e/f/g	TRAPPIST-1	~40	Multiple Earth-sized HZ planets
GJ 1002 b	GJ 1002	~16	Closest conservative HZ planet
Wolf 1069 b	Wolf 1069	~31	Earth-sized HZ orbit
TOI-700 d/e	TOI-700	~100	Two Earth-sized HZ planets
LHS 1140 b	LHS 1140	~41	Potential water world
Kepler-438 b	Kepler-438	~460	Inner edge HZ
Gliese 163 c	Gliese 163	~49	Super-Earth HZ

## Data Availability

No new data were created or analyzed in this study. Data sharing is not applicable to this article.
